# *Aspergillus oryzae* Fermentation of Lophatheri Herba Elevates SCFAs and Transforms Flavonoids to Fortify the Gut Barrier via Microbiota Remodeling in Mice

**DOI:** 10.3390/nu17182996

**Published:** 2025-09-19

**Authors:** Xin Ma, Jiaxuan Chen, Rui Chen, Wenjiao Liang, Rui Huang, Lishiyuan Tang, Lichun Qian

**Affiliations:** 1 Key Laboratory of Animal Nutrition and Feed Science in East China, Ministry of Agriculture, College of Animal Sciences, Zhejiang University, Hangzhou 310058, China; 22317062@zju.edu.cn (X.M.); 22417065@zju.edu.cn (W.L.); 22517082@zju.edu.cn (L.T.); 2 Hainan Institute of Zhejiang University, Sanya 572025, China; 22417077@zju.edu.cn (J.C.); 22317075@zju.edu.cn (R.C.); 22517069@zju.edu.cn (R.H.)

**Keywords:** *Aspergillus oryzae* fermentation, Lophatheri Herba leaves, short-chain fatty acids, flavonoid metabolites, gut microbiota

## Abstract

Background: Lophatheri Herba, a traditional East Asian herb with documented food uses, contains bioactive flavonoids. This study investigated how *Aspergillus oryzae* fermentation modifies its short-chain fatty acids (SCFAs) and metabolome, and evaluated the fermented product’s impact on intestinal barrier function in mice. Methods: Fermented leaf extracts were analyzed via GC-MS/LC-MS for SCFAs and metabolites. Forty-eight mice were divided into control (standard diet) and three experimental groups (25, 50, 100 mg/kg/day fermented product). After a 4-week intervention, duodenal morphology, colonic cytokines (IL-6/IL-1β), and cecal microbiota were assessed. Results: We identified significant SCFAs optimization. Significantly increased: acetic acid; butyric acid (*p* < 0.001); isobutyric acid (*p* < 0.01); isovaleric acid (*p* < 0.05). No significant change: propionic acid and isohexanoic acid. Significantly decreased: valeric acid and hexanoic acid (*p* < 0.001). Metabolomic remodeling showed (i) flavonoid pathway activation and (ii) key metabolite upregulation (daidzein, 4,7-dihydroxyflavone, 3,7-dimethylquercetin, aloe-emodin, soyasapogenol M1, etc.). Gut function peaked at 100 mg/kg with 18% higher duodenal villus height (*p* < 0.05), improved villus/crypt ratio, and reduced IL-6/IL-1β. Probiotic taxa including Lactobacillus, unclassified f__Lachnospiraceae, Dubosiella, and Monoglobus increased. Conclusions: Fermented Lophatheri Herba protects gut health through synergistic SCFAs optimization, flavonoid enrichment, and probiotic proliferation, supporting its potential as a microbiota-targeting functional food ingredient.

## 1. Introduction

Lophatheri Herba, a perennial bamboo-like herb from the Poaceae family, is characterized by its fast-growing nature, robustness, high regenerative capacity, and low cultivation cost. These attributes make it a highly promising natural resource for further development [1]. Additionally, it has various food-grade applications, serving as a raw material for natural antioxidants in the food industry, as well as being brewed as a herbal tea or incorporated into functional foods and beverages [2]. Lophatheri Herba’s therapeutic effects are attributed to its broad spectrum of bioactive compounds—including flavonoids, triterpenoids, phenolic acids, polysaccharides, amino acids, and trace elements—which together underpin its antibacterial, antioxidant, hypolipidemic, and cardioprotective properties [2]. Previous chromatographic characterizations of its leaves have revealed specific flavonoid glycosides: Wang Shuying et al. [3] documented luteolin, isovitexin, vitexin-4-O-glucoside, vitexin-7-O-glucoside, apigenin, and apigenin-6-C-arabinoside, while Fan et al. [4] quantified eight constituents via HPLC, notably luteolin 6-C-β-D-glucoside-(1-2)-β-D-glucopyranoside, isoquercetin, aucubin (listed twice), luteolin 6-C-β-D-glucoside-(1-2)-α-L-arabinoside, iso-rutin, luteolin 7-O-β-D-glucoside, and luteolin 6-C-α-L-arabinoside.

However, the health benefits of non-fermented Lophatheri Herba are significantly constrained by its low bioavailability [5]. Microbial fermentation enhances the bioactivity of plant materials, a finding supported by contemporary pharmacological studies. Microbial glycosidases hydrolyze flavonoid glycosides, releasing aglycones with improved bioavailability. For instance, fermented tea polyphenols demonstrate markedly greater antioxidant activity and absorption than non-fermented versions [6,7,8]. Additionally, microbial fermentation produces short-chain fatty acids (SCFAs)—key bioactive metabolites also synthesized by gut microbiota. SCFAs play pivotal roles in maintaining gut homeostasis by strengthening the intestinal barrier, modulating immune responses, and influencing host metabolism via hormone secretion [9,10]. Dietary SCFAs promote intestinal health by reshaping microbial communities, suppressing pro-inflammatory pathways, and enhancing tight junction protein expression—thereby supporting fermented Lophatheri Herba as a promising gut-health intervention [9].

Oxidative stress and inflammation represent fundamental pathological drivers in disease pathogenesis [11,12,13]. Cellular oxidative injury arises when redox imbalance surpasses endogenous protective capacity, necessitating upregulation of antioxidant defense systems—including superoxide dismutase (SOD), catalase (CAT), and glutathione peroxidase (GSH-Px)—to mitigate molecular damage [11]. Concurrently, nuclear factor kappa B (NF-κβ) serves as the master transcriptional regulator of inflammation. Pro-inflammatory cytokines (TNF-α, IL-1β, IL-6) activate NF-κβ signaling cascades, establishing feed-forward amplification loops that perpetuate inflammatory states [14,15,16]. Notably, Higa et al. [17] demonstrated that bamboo leaf extracts could inhibit the overproduction of IL-6 induced by lipotoxicity in muscle and adipocyte cell lines. Huang et al. [18] reported that bamboo leaves exhibit anti-inflammatory activity in mouse macrophages and improve inflammatory diseases. Complementing this, Jin et al. [19] documented significantly elevated SOD, GSH-Px, and CAT activity in serum and hepatic tissues alongside concurrent suppression of lipid peroxidation in serum, liver, and cerebral matrices. These findings suggest that the addition of bamboo leaf fermentation products may protect overall health by enhancing antioxidant and anti-inflammatory effects.

Dietary intervention is a key approach to regulating gut microbiota structure and function. Dietary modulation constitutes a principal strategy for optimizing gut microbiota ecology and functionality [20,21]. Dysbiosis-induced perturbations in microbial architecture and metabolic activity can trigger systemic dysfunction and pathogenesis. Fermented products demonstrate significant microbiota-remodeling capacity, exemplified by Liu et al. [22] reported that supplementation with fermented Dendrocalamus latiflorusbuds in murine models elevated GABA concentration while profoundly restructuring microbial consortia—notably enhancing α-diversity indices and enriching Bacteroidetes/Firmicutespopulations, with correlative depletion of Proteobacteriaand Actinobacteria. Xiaowei et al. [23] reported that fermented apple juice in the mouse diet effectively improved gut microbiota structure, promoting the relative abundance of beneficial bacteria such as Actinobacteria, Bifidobacterium, and Lactobacillus, while reducing oxidative damage and improving blood glucose and insulin levels. Wang et al. [24] also found that fermented Amomum villosum significantly impacted gut microbiota by increasing lactobacilli while decreasing Escherichia coli and Clostridium perfringens. Additionally, Zhao et al. [25] showed that feeding fermented foods to mice increased the relative abundance of beneficial microorganisms like Lactobacillus reuteri and Lactobacillus johnsonii in the gut.

This research evaluated *Aspergillus oryzae*-fermented Lophatheri Herba leaves for: (1) SCFAs profile modulation and secondary metabolite transformation; (2) Enteric tissue homeostasis and microbial community dynamics in murine models. The results indicate that *Aspergillus oryzae* fermentation not only increases the content of beneficial fatty acids but also enriches flavonoid compounds. The fermented extracts were associated with improved enteric health, likely through structural remodeling of the gut microbiota. Furthermore, the fermented Lophatheri Herba leaf was correlated with beneficial shifts in the composition of the gut microbiota in mice. These findings not only provide theoretical support for the high-value utilization of Lophatheri Herba leaf resources but also lay the scientific foundations for the development of a microbiota-targeting functional food ingredient.

## 2. Materials and Methods

### 2.1. Animals

Study subjects comprised 48 female C57BL/6 mice (28-day-old; Zhejiang Ziyuan source) maintained in SPF environment (20–24 °C; 40–80% RH; 12 h diurnal cycle) with free access to feed/water. Following 7-day acclimation, interventions were administered over 4 weeks per Zhejiang University Ethics Committee protocol ZJU20250737.

The mice were randomly divided into four groups, with 12 mice per group: the CON group (basic diet), T1 group (basic diet supplemented with 25 mg/kg fermented Lophatheri Herba leaf product), T2 group (basic diet supplemented with 50 mg/kg fermented Lophatheri Herba leaf product), and T3 group (basic diet supplemented with 100 mg/kg fermented Lophatheri Herba leaf product) [26]. The dosing levels (25, 50, and 100 mg/kg) were selected to assess a range of biological effects. The upper dose (100 mg/kg) was chosen based on pilot studies showing that higher doses (e.g., 200 mg/kg) reduced palatability and feed intake, which could confound the outcomes. The human equivalent dose (HED) was estimated using body surface area normalization [27], the 100 mg/kg dose in mice corresponds to approximately 8.1 mg/kg in humans, which is a feasible and safe range for a dietary supplement.

C57BL/6 mice were selected for this study due to the high similarity of their gut microbiota composition and immune system development to humans. The sample size was determined using G*Power software (v3.1.9.7) to ensure adequate statistical power. Based on this calculation, a minimum of 12 animals per group were required to achieve 80% power at a 5% significance level. To minimize confounding factors, cage location was rotated weekly across rack positions to counter environmental gradients; concurrently, blinding was implemented whereby both experimenters and data analysts were blinded to group allocation. Following 4 weeks of standardized feeding, experimental mice were fasted for 12 h (with free access to water) and then euthanized via injection of sodium pentobarbital. This euthanasia procedure strictly adhered to institutional animal ethics guidelines.

### 2.2. Fermentation of Lophatheri Herba

The *Aspergillus oryza* strain (CICC 40636) was obtained from the China Center for Industrial Microbial Culture Collection. Lophatheri Herba was sourced from local farmers in Anji County, Zhejiang Province, China, who provided the Lophatheri Herba (Phyllostachys edulis) free of charge. The Lophatheri Herba leaves were ground into powder and inoculated with Aspergillus oryza spores at a concentration of 1 × 10^7^ CFU/g. Under controlled conditions (28 ± 1 °C, 60% RH), substrates underwent 96 h fermentation prior to 60 °C dehydration and ultimate storage at −80 °C.

### 2.3. Detection of Short-Chain Fatty Acids

SCFAs—acetic acid, propionic acid, butyric acid, isobutyric acid, valeric acid, and isovaleric acid—were quantified using gas chromatography (GC; HP G1540N, Agilent Technologies, Santa Clara, CA, USA). Ferment samples (50 mg) were homogenized with 50 mL ultrapure water (18.2 MΩ·cm) via vortex oscillation (3000 rpm) for 5 min, followed by centrifugation at 10,000× *g* for 10 min. Supernatants were acidified with 25% phosphorous acid (9:1 *v*/*v*) for 3 h at 4 °C, then sterile-filtered through 0.22 μm membranes into 2 mL amber GC vials. Chromatographic separation was performed on an HP DB-FFAP capillary column (30 m × 250 μm × 0.25 μm) with ultra-pure nitrogen carrier gas (99.999%) at 0.8 mL/min and hydrogen auxiliary gas (99.999%). Detection employed a flame ionization detector (FID) at 280 °C with injection parameters: 250 °C inlet temperature, 50:1 split ratio, 1 μL injection volume. The temperature program was: 60 °C (hold 1 min) to 220 °C at 20 °C/min (hold 5 min). Calibration Standards Preparation SCFAs reference standards (Sigma-Aldrich, St. Louis, MO, USA) were dissolved in ultrapure water to prepare 1 mg/mL stock solutions. Calibration curves were constructed using working standards at concentrations of 25, 50, 100, 250, 500, and 1000 μg/mL for acetic, propionic, butyric, and isobutyric acids and 5, 10, 25, 50, 100, and 250 μg/mL for valeric and isovaleric acids. All solutions underwent identical sample pretreatment procedures prior to GC analysis.

### 2.4. Untargeted Metabolomics Analysis

Metabolite extracts were prepared for untargeted analysis by: (1) Cryo-homogenization (50 ± 5 mg sample, 400 μL methanol–water [4:1 *v*/*v*], 6 mm beads; −10 °C/50 Hz/6 min), (2) Ultrasonic-assisted extraction (40 kHz/5 °C/30 min), (3) −20 °C equilibration (30 min), (4) Centrifugation (13,000× *g*/4 °C/15 min). Clarified supernatants were cryopreserved (−80 °C) in LC-MS vials with QC cohort.

### 2.5. Hematoxylin and Eosin (H&E) Staining

Fixed in 4% PFA, duodenal/colonic tissues were paraffin-embedded, sectioned, and H&E-stained. Light microscopic examination (Olympus, Tokio, Japan, 200×/400×) enabled tissue change assessment. Quantitative morphometry (ImageJ1.54g) measured VH and CD values, with subsequent calculation of CD ratios and apparent absorption areas.

### 2.6. Ileum Antioxidant Function Assessment

Ileum tissue samples were precisely weighed (0.2 g) and mixed with nine times their weight in specific dilution buffer. Tissue samples underwent cryohomogenization (high-speed homogenizer, 60 s, ice bath) until they were particulate-free. After centrifugation (3000 rpm × 10 min), supernatants were assayed for ileal biomarkers, such as total antioxidant capacity (T-AOC), total protein (TP), glutathione S-transferase (GSH-ST) activity, and malondialdehyde (MDA) content, using Nanjing Jiancheng Bioengineering Institute kits per manufacturer protocols.

### 2.7. Immunohistochemistry Staining

Murine colon paraffin sections underwent sequential deparaffinization in xylene and rehydration through graded ethanol series (100% → 95% → 80% → 70%). Antigen retrieval was performed in 0.01 M citrate buffer (pH 6.0) at 95 °C for 20 min. Endogenous peroxidase activity was quenched with 3% H_2_O_2_ in deionized water (30 min, RT). Non-specific binding was blocked with 5% normal serum (1 h) prior to overnight incubation at 4 °C with primary anti-IL-6/IL-1β antibody (1:100 dilution). Immunoreactivity was visualized using DAB chromogen with development time optimized to prevent overstaining (positive signals: brownish yellow). Sections were counterstained with hematoxylin, dehydrated through ascending ethanol series, cleared in xylene, and mounted with resinous medium. Brightfield microscopy documented specific staining patterns.

### 2.8. Cecal Microbiota 16S rRNA Sequencing

Genomic DNA Extraction and Quality Assessment: Genomic DNA was isolated from cecal content samples using Omega Bio-tek (Norcross, GA, USA) extraction kits according to manufacturer specifications. Post-extraction quality control involved dual verification: (1) electrophoretic analysis on 1.0% (*w*/*v*) agarose gels (120 V, 30 min) to confirm DNA integrity and fragment distribution; (2) spectrophotometric quantification via NanoDrop ND-2000 (Thermo Scientific Inc., Waltham, MA, USA) to determine concentration (ng/μL) and purity (validated A_260_/A_280_ ratios of 1.8–2.0), ensuring suitability for downstream applications.

16S rRNA Gene Amplification and Processing: The V3-V4 hypervariable region of bacterial 16S rRNA genes was amplified using universal primers 341F (5′-CCTAYGGGRBGCASCAG-3′) and 806R (5′-GGACTACHVGGGTWTCTAAT-3′). PCR reactions (25 μL total volume) contained 12.5 μL of 2× Taq Master Mix, 0.5 μL of each primer (10 μM), and 50 ng template DNA. Thermal cycling parameters were initial denaturation at 95 °C for 3 min; 30 cycles of 95 °C for 30 s, 55 °C for 30 s, and 72 °C for 45 s; final extension at 72 °C for 10 min. Amplicons were electrophoretically verified on 2% agarose gels (120 V, 30 min), purified with AxyPrep DNA gel extraction kits, and quantified fluorometrically using a Qubit 3.0 instrument (Promega, Madison, WI, USA). Qualified sequencing libraries underwent paired-end sequencing (2 × 300 bp) on the Illumina MiSeq platform(Illumina, San Diego, CA, USA).

Data Processing: Raw sequencing data were first quality controlled and filtered using fastp software (v0.20.0). Dual-end reads were then accurately merged using the FLASH tool (v1.2.11). After preprocessing, sequences were further processed using the DADA2 algorithm integrated within the Qiime2 platform (2020.2 version), which constructs an error probability model for base-level correction, yielding high-confidence amplicon sequence variants (ASVs). Finally, the ASVs were classified taxonomically using the naïve Bayesian classifier in Qiime2 based on the SILVA reference database of 16S rRNA gene sequences, providing microbial community structure data from phylum to species level. Subsequent statistical analyses were then performed on these curated microbial community data. For alpha diversity group comparisons, Kruskal–Wallis tests with post hoc FDR correction were applied. Differential abundance analysis between groups was conducted using the DESeq2 method with false discovery rate (FDR) adjustment.

### 2.9. Data Analysis

Data processing utilized SPSS 20.0 for one-way ANOVA and Tukey’s HSD pairwise comparisons. Values represent mean ± SEM with statistical significance defined as *p* < 0.05. Graphical representations and analytical verification were generated using GraphPad Prism 10.1.2.

## 3. Results

### 3.1. Short-Chain Fatty Acids Detection in Fermented Lophatheri Herba Leaf Products

**Figure 1:**
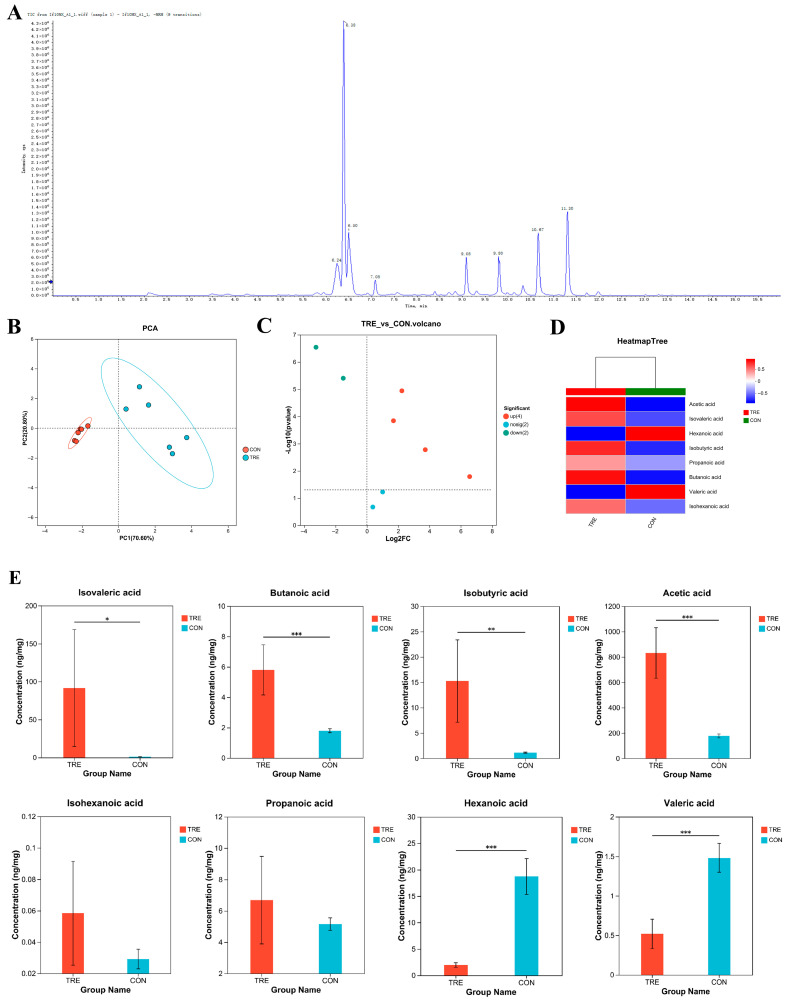
The effect of *Aspergillus oryzae* fermentation on SCFAs in Lophatheri Herba leaves; (**A**) Short-chain fatty acid TIC (Total Ion Chromatogram); (**B**) Principal Component Analysis (PCA) score plot; (**C**) volcano plot of differences between SCFAs groups; (**D**) SCFAs clustering heatmap; (**E**) quantitative composition of SCFAs; Significant differences are indicated by “*”, “**” and “***” (* *p* < 0.05, ** *p* < 0.01, and *** *p* < 0.001); CON, control group; T1, experimental group 1; T2, experimental group 2; T3, experimental group 3.

In this experiment, eight SCFAs were analyzed within 16 min. The total ion chromatogram (TIC) showed high separation and good peak shapes for all indicators (Figure 1A). To investigate the effects of *Aspergillus oryzae* fermentation on Lophatheri Herba leaves, non-targeted metabolomic analysis based on LC-MS/MS was employed to quantify the SCFAs in fermented Lophatheri Herba leaf products. Figure 1B illustrates PCA results where PC1 (70.6%) and PC2 (20.8%) jointly explained 91.4% of dataset variance (Figure 1B).

A volcano plot comparing the control group and the fermented Lophatheri Herba leaf group is shown in Figure 1C. We identified four significantly upregulated SCFAs, two significantly downregulated metabolites, and two metabolites with no significant difference. Hierarchical clustering analysis (Figure 1D) demonstrated distinct metabolite expression profiles relative to controls. Significant elevations were observed in isobutyric acid, isovaleric acid, acetic acid, and butyric acid levels, while isohexanoic acid and propionic acid exhibited reduced expression.

Complementary quantitative analysis (Figure 1E) illustrates group-specific metabolite abundance through bar charts, with the x-axis denoting experimental/control groups and the y-axis representing normalized mass spectrometry intensity values. Error bars indicate mean ± standard deviation, with asterisks denoting statistical significance (*p* < 0.05, *p* < 0.01, *p* < 0.001). The results indicated that the levels of acetic acid and butyric acid in the fermented Lophatheri herb leaf group were significantly higher than those in the control group (*p* < 0.001). Among them, isobutyric acid increased highly significantly (*p* < 0.01), and isovaleric acid increased significantly (*p* < 0.05).No significant differences were observed in the levels of isohexanoic acid and propionic acid in the experimental samples compared to the control group. In contrast, valeric acid and hexanoic acid were significantly reduced, and the differences were statistically significant (*p* < 0.001).The absolute concentrations of acetic acid, propionic acid, butyric acid, isobutyric acid, isovaleric acid, and isohexanoic acid increased by 654.64, 1.53, 4.01, 14.11, 90.57, and 0.03 ng/mg, respectively, while the absolute concentrations of valeric acid and hexanoic acid decreased by 0.96 and 0.96 ng/mg, respectively.

### 3.2. Metabolomics of Fermented Lophatheri Herba Leaves

**Figure 2:**
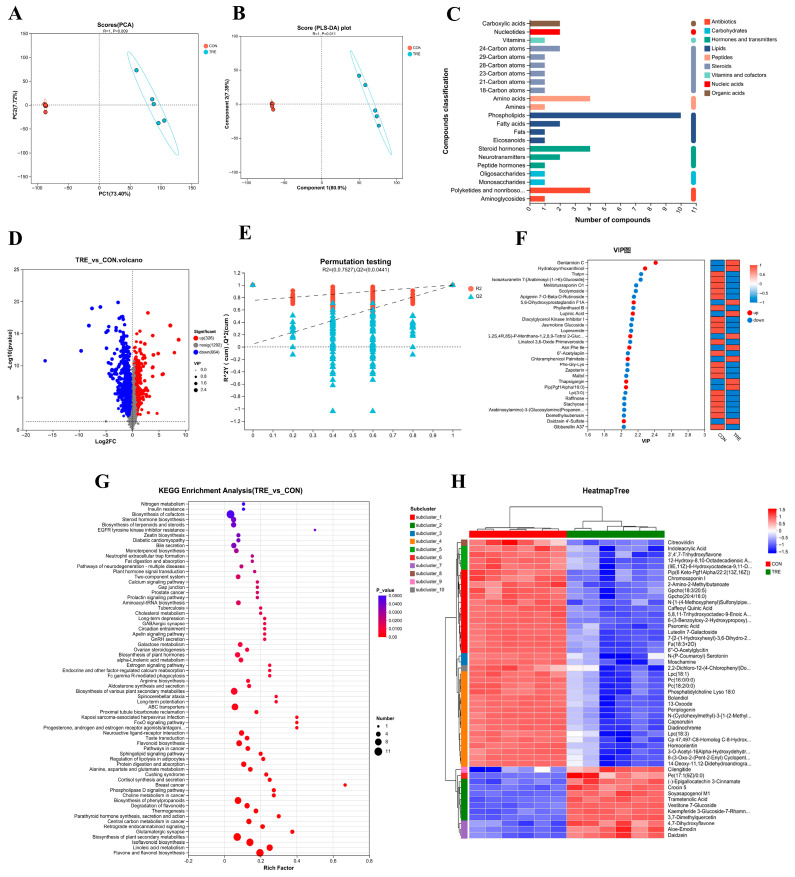
Metabolomics of Fermented Lophatheri Herba Leaf Products. (**A**) Principal component analysis (PCA) score plot of untargeted metabolite profiles; (**B**) partial least squares discriminant analysis (PLS-DA) score plot between groups; (**C**) KEGG classification of differential metabolites; (**D**) volcano plot of differential metabolites; (**E**) PLS-DA cross-validation test; (**F**) VIP value analysis of the top 30 differential metabolites; KEGG classification of 32 differential metabolites; (**G**) KEGG enrichment bubble plot for differential metabolites; (**H**) heatmap of differential metabolites between groups (*p* < 0.05);CON, control group; T1, experimental group 1; T2, experimental group 2; T3, experimental group 3.

Multivariate analysis revealed significant group separations across methodologies (Figure 2). Principal Component Analysis demonstrated that PC1 and PC2 collectively accounted for 81.12% of cumulative variance (73.4% and 7.72%, respectively). Complementary PLS-DA modeling of mixed-ion data showed distinct clustering (R = 1, *p* = 0.011), with the first component explaining 80.9% variance and the second contributing 7.39%. These results collectively confirm pronounced metabolic profile distinctions between experimental and control cohorts.

The results from the PLS-DA permutation test demonstrated a decline in R^2^ and Q^2^ values as the permutation retention decreased, with the regression line showing an upward trend. The Q^2^ regression line’s intercept with the y-axis was less than 0.05, confirming the robustness of the model without overfitting and indicating that it accurately reflects the sample data (Figure 2E).

In this study, a total of 990 differential metabolites were identified between the experimental and control groups, with 326 metabolites upregulated and 664 downregulated (Figure 2D). The metabolite abundance heatmap (top 50 ranked metabolites) demonstrates distinct expression patterns between experimental and control cohorts. Quantitative analysis revealed 12 significantly upregulated metabolites and 38 downregulated metabolites relative to controls (Figure 2H).

To further investigate the impact of fermentation on Lophatheri Herba leaves, a KEGG pathway analysis was conducted on the 990 differential metabolites, with annotation of relevant metabolic pathways. Significantly enriched metabolic pathways (*p* < 0.05) differentiating experimental and control groups are visualized in Figure 2G, where bubble size corresponds to the number of enriched compounds per pathway. The experimental cohort exhibited marked enrichment in 17 key pathways including cofactor biosynthesis, flavonoid pathways (flavone/flavonol, isoflavonoid, degradation), plant secondary metabolism (general, phenylpropanoids, terpenoids/steroids, hormones), lipid metabolism (linoleic acid), transport systems (ABC transporters), cancer metabolism (central carbon), protein digestion/absorption, neuroactive ligand-receptor interactions, bile secretion, and steroid hormone biosynthesis

Furthermore, Figure 2C depicts the KEGG compound classification of the 990 differential metabolites, identifying 10 types of phospholipids, including Pe(14:0/22:5), Gpetn(14:0/22:6), Gpcho(18:3/20:5), Gpcho(20:4/16:0), Gpetn(14:0/22:4), Pe(14:0/15:0), Pe(14:0/16:0), Pe(14:0/22:6), Lpc(18:3), and Pc(14:0/20:1), as well as four steroid hormones, namely Beta-Estradiol, Progesterone, 17-Hydroxyprogesterone, and Prasterone Sulfate. Additionally, four polyketides and nonribosomal peptides, including Erythromycin, Geldanamycin, Oleandomycin, and Monensin, were identified. Other metabolites such as L-Glutamine, L-Asparagine, L-Aspartic Acid, and Glutamic Acid were also categorized as amino acids.

Finally, Figure 2F displays an analysis of the top 30 metabolites with VIP ≥ 1, revealing significant upregulation of metabolites such as Gentamicin C, Hydratopyrrhoxanthinol, 5,6-Dihydroxyprostaglandin F1A, Lupinic Acid, (1S,2S,4R,8S)-P-Menthane-1,2,8,9-Tetrol 2-Glucoside, Asn Phe Ile, Chloramphenicol Palmitate, Thapsigargin, Pip(Pgf1Alpha/16:0), and Daidzein 4′-Sulfate in the experimental group.

### 3.3. Impact of Fermented Products on Mouse Growth Performance

**Figure 3:**
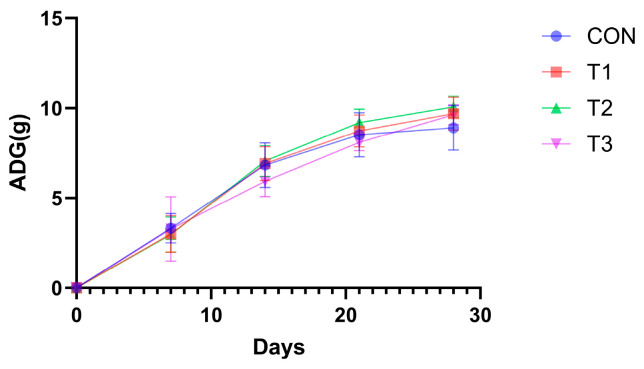
Effect of fermented Lophatheri Herba leaf products on mouse body weight. Note: ADG: Average Daily Gain.

Murine weight dynamics (Figure 3 and Table 1) revealed significantly greater gains (*p* < 0.05) in the experimental groups versus controls during all measured intervals (0–14 d, 0–21 d, 0–28 d), with group T2 showing maximal increase. Furthermore, the mean initial body weight was 9.62 ± 0.23 g.

### 3.4. Effect of Fermented Lophatheri Herba Leaves on Intestinal Tissues of Mice

**Figure 4:**
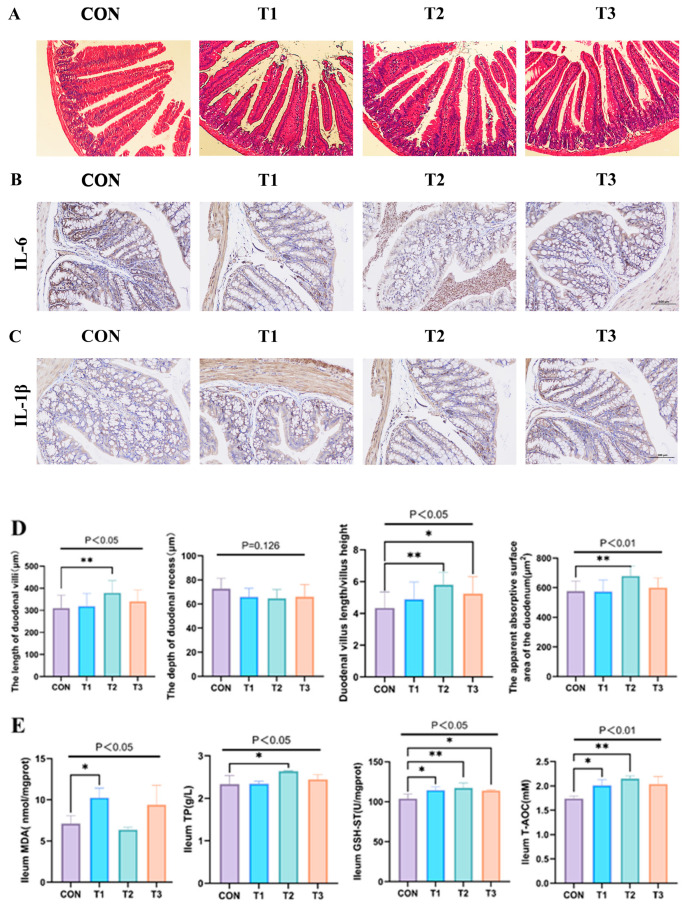
Illustration of the effects of fermented Lophatheri Herba leaf products on the intestinal tissues of mice. Panel (**A**) presents the morphological analysis of duodenal tissue at 100× magnification. Panels (**B**,**C**) show the immunohistochemical staining results of IL-6 and IL-1β in the colonic tissue of mice, respectively, at 200× magnification. Panel (**D**) includes measurements of villus height, crypt depth, the ratio of villus height to crypt depth, and the apparent absorption area in the duodenum. Finally, panel (**E**) demonstrates the effects of the fermented Lophatheri Herba leaf products on the antioxidant capacity in the ileum, as assessed by MDA, TP, GSH-ST, and T-AOC levels. Significant differences are indicated by “*” and “**” (* *p* < 0.05, and ** *p* < 0.01); The groups are as follows: CON, control group; T1, experimental group 1; T2, experimental group 2; T3, experimental group 3.

Histomorphological assessment of intestinal tissues via H&E staining (Figure 4A) revealed significant alterations in duodenal architecture following fermented Lophatheri Herba intervention. As shown in Figure 4D, the duodenal villus height was significantly increased by 18% in the 50 mg/kg dose group (Test Group 2: 378.06 ± 56.42 μm) compared to the control group (316.07 ± 60.76 μm) (*p* < 0.05). The absolute mean difference was 61.99 μm, with a very large effect size (Cohen’s *d* = 1.06). No statistically significant differences were observed between the control and other dose groups. Additionally, compared to controls (CON), the T2 group exhibited an unchanged crypt depth (CD; NS), an elevated VH/CD ratio (*p* < 0.05), and an expanded apparent absorption area (*p* < 0.01). These structural modifications indicate enhanced absorptive surface development in the duodenum (Figure 4D).

Further analysis of antioxidant-related capacities in the duodenum indicated significant differences in GSH-ST and TP levels between the experimental and control groups (*p* < 0.05), with the experimental group showing a significant increase. Oxidative Stress Modulation: Experimental groups exhibited increased T-AOC (*p* < 0.01) and decreased MDA (*p* < 0.05) relative to controls (Figure 4E).

Cytokine Expression: Immunohistochemistry demonstrated attenuated IL-6 and IL-1β immunoreactivity in intervention groups, with maximal reduction in T2 cohort (200× magnification; Figure 4B,C).

### 3.5. Effects of Fermented Lophatheri Herba Leaf Products on the Microbiota of Mouse Cecal

Alpha diversity metrics were employed to characterize cecal microbial communities: richness indices: ACE, Sobs, and Chao (higher values = greater species richness); evenness metric: Shannon index (higher values = more uniform species distribution); diversity index: Simpson (lower values = higher diversity).

**Figure 5:**
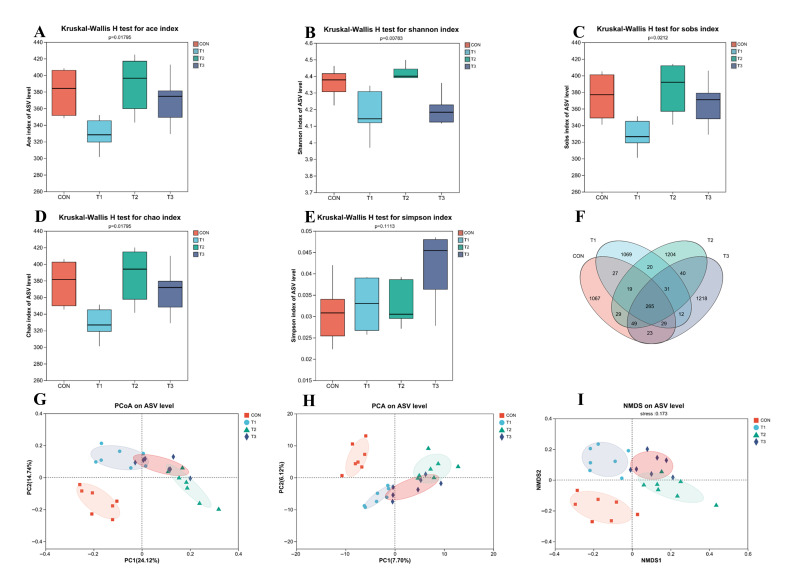

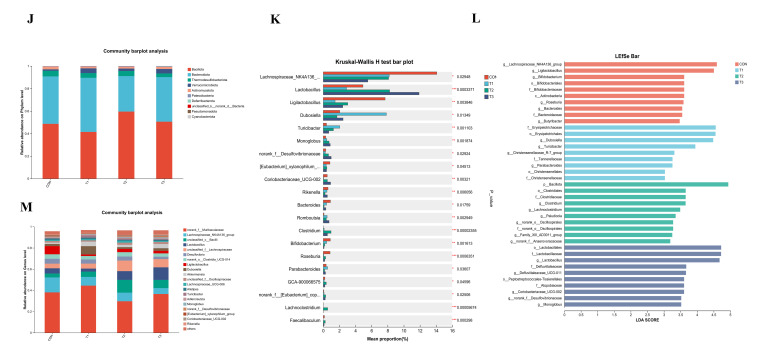
Impact of fermented Lophatheri Herba leaf products on murine cecal microbiota; (**A**–**E**) Alpha diversity indices: (**A**): ACE richness estimator, (**B**): Shannon evenness index, (**C**): Observed species (Sobs), (**D**): Chao1 richness estimator, (**E**): Simpson diversity index; (**F**) Venn diagram of amplicon sequence variants (ASVs);(**G**–**I**) Beta diversity analyses (Bray–Curtis distance): (**G**): Principal Coordinate Analysis (PCoA), (**H**): Principal Component Analysis (PCA); (**I**): Non-metric Multidimensional Scaling (NMDS); (**J**) Phylum-level taxonomic composition; (**K**) top 10 differentially abundant genera (mean relative abundance); (**L**) LEfSe analysis histogram; (**M**) genus-level taxonomic composition; Significant differences are indicated by “*”, “**” and “***” (* *p* < 0.05, ** *p* < 0.01, and *** *p* < 0.001); CON, control group; T1, experimental group 1; T2, experimental group 2; T3, experimental group 3.

Statistical comparisons revealed the following: no significant differences in species richness indices (ACE/Sobs/Chao; *p* > 0.05) between the treatment (T1–T3) and control (CON) groups (Figure 5A,C,D), Significantly elevated Shannon index in the treatment groups (*p* < 0.01) versus CON (Figure 5B)

There was a comparable Simpson diversity across all groups (*p* > 0.05) (Figure 5E). Venn diagram analysis identified a total of 5102 Amplicon Sequence Variants (ASVs) across all groups. The number of unique ASVs in each group was 1067 (CON), 1069 (T1), 1204 (T2), and 1218 (T3). A core set of 265 ASVs was shared among all four groups (Figure 5F). Beta diversity analysis demonstrated distinct clustering patterns. Principal Coordinates Analysis (PCoA), Principal Component Analysis (PCA), and Non-metric Multidimensional Scaling (NMDS) plots indicated substantial overlap among the three treatment groups (T1, T2, T3) (Figure 5G–I). Furthermore, the microbial communities in the treatment groups exhibited a divergent trend from those of the control group along the respective ordination axes.

Microbial biomarkers were identified through LEfSe analysis, revealing 63 significantly differential taxa across four groups using the top 20 features/group criterion (Figure 5L. Key differentiating taxa belonged primarily to the phyla Bacillota and Deferribacterota, and included various classes and orders such as Actinobacteria, Bifidobacteriales, Erysipelotrichales, Clostridiales, Lactobacillales, Monoglobales, Christensenellales, Peptostreptococcales-Tissierellales, and Oscillospirales.

Phylum-level community profiling (Figure 5J) revealed Bacillota and Bacteroidota as dominant taxa. Specifically, the experimental groups exhibited elevated Bacillota abundance relative to controls. At the genus level, the primary genera identified include norank_f__Muribaculaceae, Chunospiraceae_NK4A136_group, unclassified_c__Bacilli, Lactobacillus, unclassified_f__Lachnospiraceae, Desulfovibrio, norank_o__Clostridia_UCG-014, Ligilactobacillus, Dubosiella, and Akkermansia (Figure 5M).

Genus-level profiling identified 10 taxa with both significant differential abundance (*p* < 0.05) and the highest mean abundance in the experimental groups versus CON (Figure 5K). Enriched genera included Lactobacillus, unclassified Lachnospiraceae(f), unclassified Bacilli(c), Dubosiella, Turicibacter, Monoglobus, Coriobacteriaceae_UCG-002, Romboutsia, Clostridium, and GCA-900066575.

## 4. Discussion

*Aspergillus oryzae* fermentation of Lophatheri Herba leaves significantly altered short-chain fatty acid composition. Relative to unfermented controls, fermented samples exhibited significantly increased acetic acid, butyric acid (*p* < 0.001); isobutyric acid (*p* < 0.01); isovaleric acid (*p* < 0.05) and no significant change in propionic acid and isohexanoic acid. Valeric acid and hexanoic acid significantly decreased (*p* < 0.001). These observed shifts in SCFAs composition may contribute to intestinal homeostasis, as SCFAs are known to influence key physiological processes including epithelial barrier function, immunomodulation, and microbial ecology. Butyrate, as the primary energy substrate for colon epithelial cells, helps preserve mucosal barrier integrity by promoting cell metabolism and differentiation [28]. Acetate and propionate are delivered to hepatocytes via the portal circulation, serving as substrates for hepatic gluconeogenesis and modulators of systemic metabolic homeostasis. However, at high concentrations, some SCFAs can have adverse effects: valeric acid, when exceeding 0.5% of total SCFAs, is significantly associated with undesirable sensory properties of fermentation products (e.g., rancid odors) and can inhibit epithelial cell viability [29]. Supraphysiological hexanoic acid levels induce inflammatory responses through expansion of Th1/Th17 lymphocyte subsets and their immunogenic byproducts [30]. In this study, fermentation by *Aspergillus oryzae* reduced valeric acid and hexanoic acid concentrations by 65% and 90%, respectively, effectively demonstrating the functional optimization of SCFAs composition through fermentation.

Experimental evidence confirms SCFAs as pivotal regulators of gut homeostasis. These metabolites modulate colonic epithelial and immune cell proliferation, differentiation, gene expression, and inflammatory responses through anti-inflammatory pathways [14,15,16,31]. Butyrate exemplifies this mechanism: by binding GPR43 receptors, it suppresses pro-inflammatory mediators (iNOS, COX-2, IL-6), as demonstrated in Svenningsen et al.’s research [32]. A. Andoh et al. [33] found that the addition of SCFAs decreased IL-8 expression, exerting an anti-inflammatory effect. Y. Zhang et al. [34] demonstrated that SCFAs significantly suppressed the expression of pro-inflammatory cytokine IL-1β and notably improved cellular oxidative stress. Based on these theoretical foundations, this study hypothesizes that SCFAs derived from fermented Lophatheri Herba leaves may protect gut health by reducing the production of inflammatory factors. Immunohistochemical analysis confirmed attenuated IL-6 and IL-1β immunoreactivity in experimental cohorts, with maximal reduction observed in Group 2.

KEGG pathway analysis of the differential metabolites in fermented Lophatheri Herba leaves revealed significant enrichment of several flavonoid-related pathways (*p* < 0.05 and bubble size > 5), including the biosynthesis of flavonoids and flavonols, isoflavonoid biosynthesis, flavonoid biosynthesis, and flavonoid degradation. These findings suggest that *Aspergillus oryzae* fermentation significantly altered the flavonoid composition in Lophatheri Herba leaves. Heatmap visualization of metabolomic profiles demonstrated significant upregulation of numerous metabolites in treatment groups versus controls. These upregulated metabolites include Daidzein, Aloe-Emodin, 4,7-Dihydroxyflavone, 3,7-Dimethylquercetin, Kaempferide 3-Glucoside-7-Rhamnoside, Vestitone 7-Glucoside, Trametenolic Acid, Soyasapogenol M1, Crocin 5, (-)-Epigallocatechin 3-Cinnamate, Pe(17:1(9Z)/0:0), and Cilengitide. Notably, these upregulated metabolites may exert synergistic effects in promoting gut health.

Daidzein, a soy-derived isoflavonoid, shows considerable potential in food and pharmaceutical applications [35,36]. S. Guo et al. demonstrated that daidzein supplementation reduces IL-6 and IL-8 levels, supporting overall health [37]. Fermentation of soybean pomace was shown by S. Gupta et al. to elevate levels of daidzein, quercetin, and puerarin [38], while C. Meng et al. reported increased isoflavones including kaempferol, quercetin, and daidzein via microbial fermentation [39]. Y.H. Lee et al. further confirmed that *Aspergillus oryzae* fermentation enhances metabolites such as daidzein and puerarin while reducing carbohydrate content, consistent with our results [40]. Aloe-emodin (AE), a hydroxyl-anthraquinone from Aloe vera, exhibits specific anti-neuroectodermal tumor activity in vitro and in vivo, with low toxicity in murine models, highlighting its promise as an anti-tumor lead compound [41]. A.A. Abdellatef et al. reported that AE inhibits NF-κβ activity and downstream target expression [42]. H. Xie et al. found that microbial fermentation increased anthraquinone compounds including aloe-emodin, potentially enhancing gut health [43].

4,7-Dihydroxyflavone, a bioactive flavonoid, competitively inhibits COX-1/COX-2. X. Li et al. identified its role in inducing ROS-mediated apoptosis in Leishmania parasites [44], suggesting anti-Leishmania potential. T. Qu et al. proposed that it modulates biological processes via inhibition of NF-κβ and HIF-1 pathways, and may overcome EGFR tyrosine kinase inhibitor resistance [45].

3,7-Dimethylquercetin and kaempferol 3-glucoside-7-rhamnoside, both flavonol derivatives, support gut health by modulating inflammatory pathways through restrain IL-1β, IL-6, iNOS, and COX-2 expression [46,47,48]. S. Claude further validated the anti-inflammatory effects of flavonols [49]. Trametenolic acid (TA), a triterpenoid from Inonotus obliquus, suppresses TNF-α, IL-6, and IL-1β, activates Nrf2 signaling, and upregulates HO-1 and NQO-1, demonstrating gut-protective potential through antioxidant and anti-inflammatory mechanisms [49].

Soyasapogenol M1, a soyasapogenol subtype, was reported by H.-J. Lee et al. to alleviate intestinal inflammation and improve gut microbiota in mice [50]. Z. Khaksari et al. showed that Crocin 5 reduces TNF-α, IL-1β, NF-κβ mRNA, and ROS generation [51], consistent with our findings on bamboo leaf fermentation products. S. Bharrhan et al. indicated that catechins and quercetin inhibit TNF-α and NO expression [52]. (-)-Epigallocatechin 3-cinnamate, a flavonoid derivative, exhibits anticancer properties [53], while (+)-catechins inhibit monocyte adhesion to IL-1β-stimulated endothelial cells [54].

Cilengitide, an Arg-Gly-Asp cyclic pentapeptide, blocks integrin activation via αvβ3 and αvβ5, suppresses integrin overexpression in malignancies, and shows antitumor efficacy in glioblastoma models. It also sensitizes tumors to radiation and chemotherapy [55].

Therefore, the metabolites from the Lophatheri Herba leaf fermentation products, including Daidzein, Aloe-Emodin, 4,7-Dihydroxyflavone, 3,7-Dimethylquercetin, Kaempferide 3-Glucoside-7-Rhamnoside, Trametenolic Acid, Soyasapogenol M1, and Crocin 5, may synergistically contribute to the positive effects on overall health.

Quantitative analysis of cecal microbiota identified ten genera with significantly elevated abundance in treatment groups relative to CON: Lactobacillus, unclassified Lachnospiraceae(f), unclassified Bacilli(c), Dubosiella, Turicibacter, Monoglobus, Coriobacteriaceae_UCG-002, Romboutsia, Clostridium, and GCA-900066575.

Lactobacillus, a well-known probiotic, contributes significantly to food digestion, nutrient absorption, pathogen defense, inflammation regulation, gut microbiota maintenance, and prevention of bacterial infections [56,57]. Specifically, Cheng et al. [58] demonstrated that L. gasseri JM1 enhances SCFAs biosynthesis and suppresses expression of TNF-α, IL-1β, and IL-6 in colonic tissue. The study also found that it promotes ERK phosphorylation, thereby reducing cell necrosis. Notably, unclassified_f_Lachnospiraceae, a member of the Lachnospiraceae family, participates in SCFAs metabolism such as butyrate production, which alleviates inflammation through modulation of signaling pathways and improves gut health [59,60]. Supplementation with Artemisia vulgaris extract was shown to increase its abundance, ameliorating gut dysbiosis [61]. Similarly, Chen et al. [62] reported that L-malic acid supplementation markedly elevated unclassified_f__Lachnospiraceae levels, potentially contributing to antioxidant and anti-inflammatory effects.

At the genus level, unclassified_c__Bacilli is also recognized as a beneficial bacterium. Li et al. [63] observed that oral nanodrug treatment for rectal cancer increased the abundance of beneficial bacteria including Bacillus and unclassified_c__Bacilli, leading to improved overall health. Similarly, the rise in Dubosiella abundance has been linked to improved host metabolism and immune regulation in several studies [64]. For instance, Zhang et al. [65] demonstrated that apple polyphenol supplementation led to a concurrent increase in beneficial bacteria, including Dubosiella. Furthermore, Li et al. [64] and Mo et al. [66] also reported positive associations between Dubosiella and markers of gut health following different dietary interventions. While these consistent observations across studies suggest a potential beneficial role, the specific causal contributions of Dubosiella remain to be fully elucidated.

Research indicates that Turicibacter influences lipid homeostasis by modulating serum lipid profiles; for example, it reduces triglycerides in mice [67]. Liu et al. [68] showed that Fucoidan increases the abundance of Lachnospiraceae bacteria—including Turicibacter, Muribaculum, Parasutterella, and Colidextribacter—promoting SCFAs production (particularly butyrate). This consequently suppresses pro-inflammatory cytokines (IL-1β and IL-6), alleviates colitis, and enhances gut barrier function. Similarly, Xu et al. [69] reported that Inulin supplementation elevates Turicibacter levels, potentially improving intestinal barrier integrity, reducing neuroinflammation, and preserving neuronal function.

The increase in Monoglobus may be of functional relevance, as members of this genus have been associated with the production of SCFAs in previous studies [70]. This is supported by the findings of Xie, F. et al. [71], who observed that an increase in Monoglobus was correlated with the alleviation of ulcerative colitis in mice. However, the exact mechanistic role of this genus in host health requires further investigation.

Although Coriobacteriaceae_UCG-002 belongs to the phylum Actinobacteria, it has been associated with improved insulin sensitivity [72]. Gao et al. [73] demonstrated that EPA/DHA-PC supplementation enriched beneficial taxa such as Bacteroidetes, Akkermansia, Lactobacillus, Coriobacteriaceae_UCG-002, and Clostridium_sensu _stricto_1, thereby ameliorating gut dysbiosis. Wu, Y. et al. [74] further showed that modulation of gut microbes—including Lactobacillus, Muribaculum, Candidatus_Stoquefichus, and Coriobacteriaceae_UCG-002—influences tryptophan and linoleic acid metabolism, reducing inflammation and oxidative stress while promoting neuronal health. Additionally, Song et al. [75] reported that Romboutsia enhances commensals such as Enterococcus and Pseudomonas in broilers, contributing to immune reinforcement.

As keystone commensals, Clostridium species help maintain intestinal homeostasis through immunomodulation and SCFAs production. They alleviate inflammation and allergic diseases, while metabolites like butyrate, secondary bile acids, and indolepropionic acid reinforce the epithelial barrier and interact with the immune system [76]. Certain Clostridium strains also exhibit potential in cancer therapy and targeted delivery of oncolytic viruses. Moreover, the abundance of GCA-900066575 has been significantly increased in studies. For instance, Guo, J. et al. [77] observed that hydroxyphenylpropionic acid raised the levels of GCA-900066575 and unclassified_Lachnospiraceae. Similarly, Chen, Y. et al. [78] found that Dendrobium officinale supplementation increased the abundance of Enterobacter, GCA_900066575, Muribaculum, and Bacteroides.

In summary, this study demonstrates that the fermented Lophatheri Herba extract significantly improves intestinal barrier function and overall gut health. Our findings reveal that *Aspergillus oryzae* fermentation optimizes the profile of SCFAs and flavonoids, and enriches beneficial microbiota, collectively contributing to the observed anti-inflammatory and barrier-protective effects. Although the sample size was statistically adequate to detect primary outcomes with high power, we acknowledge that future studies with larger cohorts would enhance the robustness of the data and allow analysis of secondary variables. It should also be noted that the absence of a non-fermented control group limits our ability to fully attribute the effects solely to fermentation, though prior literature suggests that fermentation markedly enhances bioactive compound profiles and sensory properties compared to the raw herb [79]. Furthermore, while the four-week intervention was sufficient to reveal significant acute benefits, longer studies are needed to evaluate long-term efficacy and safety.

Regarding the observed bioactivity, although we detected strong associations between increased SCFAs/flavonoids and functional improvements, these correlations should be interpreted as generating mechanistic hypotheses. Therefore, further targeted interventions—such as metabolite supplementation or genetic manipulations—are required to validate causal roles. Finally, while murine models provide compelling preclinical evidence, well-designed clinical trials are essential to confirm translatability to humans. In this context, the dosage and microbial biomarkers identified here offer a rational starting point for such trials.

On a mechanistic level, preliminary data (Supplementary Figure S1) suggest that the extract may inhibit IKK activation within the NF-κβ pathway. Nevertheless, intestinal health likely involves synergistic regulation across multiple signaling networks. Thus, future studies using chemical inhibitors or gene knockout models.

## 5. Conclusions

Our study demonstrates that *Aspergillus oryzae* fermentation serves as a potent food-grade bioprocessing strategy to unlock the latent bioactivity of Lophatheri Herba leaves, culminating in gut health optimization through integrated synergistic mechanisms: First, the SCFAs profile is functionally reshaped, with the contents of acetate, acetic acid; butyric acid (*p* < 0.001); isobutyric acid (*p* < 0.01); and isovaleric acid (*p* < 0.05), being significantly increased. Propionic acid and isohexanoic acid experienced no significant change. Valeric acid and hexanoic acid (*p* < 0.001) significantly decreased. Second, anti-inflammatory metabolites target the activation of flavonoid biosynthesis pathways, leading to the enrichment of metabolites like daidzein, aloe-emodin, 4,7-dihydroxyflavone, 3,7-dimethylquercetin, kaempferide 3-glucoside-7-rhamnoside, vestitone 7-glucoside, trametenolic acid, soyasapogenol M1, crocin 5, (-)-epigallocatechin 3-cinnamate, Pe(17:1(9Z)/0:0), and cilengitide, which may suppress the production of pro-inflammatory factors. Third, enhanced gut function was universal, peaking in experimental group 2 as evidenced by an 18% rise in duodenal villus height (*p* < 0.05), concomitant augmentation of the villus-to-crypt (V/C) depth ratio, and reduced expression of IL-6/IL-1β cytokines in the colon. Synergistic microbiota interactions elevate abundance of homeostasis-promoting taxa: Lactobacillus, unclassified Lachnospiraceae, unclassified Bacilli, Dubosiella, Turicibacter, and Monoglobus. This study suggests that fermented Lophatheri Herba leaves may contribute to gut health through associated changes in SCFAs profile, flavonoid enrichment, and probiotic abundance, thereby highlighting the potential of fermented plant-based substrates in gut health regulation. Further research is warranted to fully elucidate the underlying mechanisms.

## Figures and Tables

**Table 1:** Effect of fermented Lophatheri Herba leaf products on mouse body weight.

Items	CON	T1	T2	T3	SEM	*p*
0–7 d ADG	3.32	3.00	2.97	3.28	0.17	0.839
0–14 d ADG	6.83	6.92	7.06	5.94	0.15	0.041
0–21 d ADG	8.52	8.73	9.19	8.10	0.14	0.046
0–28 d ADG	8.91	9.70	10.07	9.63	0.15	0.044
FCR (F/G)	14.60	11.49	10.46	11.63	0.33	<0.001

Note: ADG: Average Daily Gain; SEM, standard error of the mean; CON, control group; T1, experimental group 1; T2, experimental group 2; T3, experimental group 3.

## Data Availability

All data generated or analyzed during this study are included in the published article. Additional supporting data are available in the OSF repository at [https://doi.org/10.17605/OSF.IO/CJGAE].

## Supplementary Materials

The following supporting information can be downloaded at: https://www.mdpi.com/article/10.3390/nu17182996/s1, Figure S1: Western blot images of P65, IKK and IBA proteins in the colon tissue of mice and the ratio of gray values of the detected protein bands to those of the internal reference protein.
