# Supplementary material for: Aspergillus oryzae Fermentation of Lophatheri Herba Elevates SCFAs and Transforms Flavonoids to Fortify the Gut Barrier via Microbiota Remodeling in Mice
Source: Nutrients. 2025 Sep 19;17(18):2996. doi: 10.3390/nu17182996 (PMC12472607; doi:10.3390/nu17182996)
Supplement: Supplementary file 1 [file nutrients-17-02996-s001.zip › nutrients-3854798-Supplementary Material.pdf]

Supplementary material

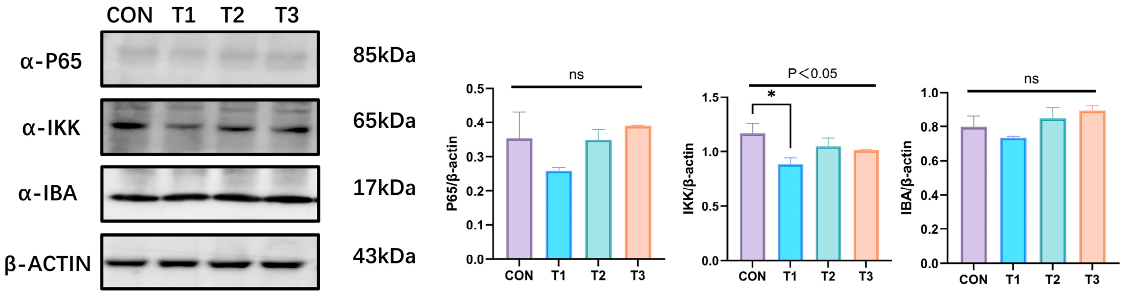

Figure S1. Western blot images of P65, IKK and IBA proteins in the colon tissue of mice and the ratio of gray values of the detected protein bands to those of the internal reference protein.
